# Sex Determination of Human Nails Based on Attenuated Total Reflection Fourier Transform Infrared Spectroscopy in Forensic Context

**DOI:** 10.3390/s23239412

**Published:** 2023-11-26

**Authors:** Bilkis Mitu, Václav Trojan, Lenka Halámková

**Affiliations:** 1 Department of Environmental Toxicology, Texas Tech University, Lubbock, TX 79409, USA; bilkis.mitu@ttu.edu; 2 Cannabis Facility, International Clinical Research Centre, St. Anne’s University Hospital, 602 00 Brno, Czech Republic; vaclav.trojan@fnusa.cz; 3 Department of Natural Drugs, Faculty of Pharmacy, Masaryk University, 612 00 Brno, Czech Republic

**Keywords:** ATR FT-IR spectroscopy, machine learning, artificial neural network (ANN), partial least-square discriminant analysis (PLS-DA), male, female

## Abstract

This study reports on the successful use of a machine learning approach using attenuated total reflectance Fourier transform infrared (ATR FT-IR) spectroscopy for the classification and prediction of a donor’s sex from the fingernails of 63 individuals. A significant advantage of ATR FT-IR is its ability to provide a specific spectral signature for different samples based on their biochemical composition. The infrared spectrum reveals unique vibrational features of a sample based on the different absorption frequencies of the individual functional groups. This technique is fast, simple, non-destructive, and requires only small quantities of measured material with minimal-to-no sample preparation. However, advanced multivariate techniques are needed to elucidate multiplex spectral information and the small differences caused by donor characteristics. We developed an analytical method using ATR FT-IR spectroscopy advanced with machine learning (ML) based on 63 donors’ fingernails (37 males, 26 females). The PLS-DA and ANN models were established, and their generalization abilities were compared. Here, the PLS scores from the PLS-DA model were used for an artificial neural network (ANN) to create a classification model. The proposed ANN model showed a greater potential for predictions, and it was validated against an independent dataset, which resulted in 92% correctly classified spectra. The results of the study are quite impressive, with 100% accuracy achieved in correctly classifying donors as either male or female at the donor level. Here, we underscore the potential of ML algorithms to leverage the selectivity of ATR FT-IR spectroscopy and produce predictions along with information about the level of certainty in a scientifically defensible manner. This proof-of-concept study demonstrates the value of ATR FT-IR spectroscopy as a forensic tool to discriminate between male and female donors, which is significant for forensic applications.

## 1. Introduction

Forensic Onychology (Greek Onuks = nail; Logia = study of), the study of fingernails and toenails, has the potential to offer crucial insights to determine the individuality of a person in criminal investigation [1]. Human phenotype profiling comprises a set of critical characteristics that can be used to narrow down a potential suspect list against a crime [2]. During the last few decades, both fingernails and toenails have become very beneficial specimens for the detection of chemical substances, toxic materials, or drug use and abuse [3,4,5]. Nails serve as valuable tissue specimens for human identification as they retain discrete details of genetic information [1,6,7]. Fingernails are a reservoir of keratin fibers for catching and trapping drugs [1,5,7], alcohol biomarkers [5], diet [1], explosive residues [1,6], or various pollutants. Conventionally, blood and serum are the most common and useful specimens for phenotyping, diagnosis, and drug detection [8]. The increasing popularity of screening programs has led to a need for alternative human tissue specimens that exhibit adequate sensitivity and specificity in detecting their intended target. Nails have become an excellent alternative tissue specimen due to their low cost, easy storage and transportation, sample size and sample process, and non-invasive and non-destructive nature [8,9,10,11].

Chemically, human fingernails are composed of proteins (keratin), lipids (mostly cholesterol), minerals (Cl, Ca, K, Na, Si, Mg, Zn, Fe, Al, Br, Cu), and water [12,13,14]. Nails primarily consist of hard alpha-keratin, which is a fibrous protein. Environmental exposure and genetic determinants [12,15,16,17] can influence trace elements and their composition in nails. So far, research has focused only on a quantitative analysis of these organic and inorganic chemical composition levels to differentiate gender/sex. In 2000, Rodushkin et al. [16] studied elemental characterization to discriminate between male and female. They revealed that males have higher levels of Na, K, and Mn and lower levels of bismuth and silicon in comparison to females. Park and Kwon [15] conducted a comparative analysis of the major mineral content between males and females. The study revealed that males exhibit higher levels of nitrogen (N) and potassium (K) in comparison to females, while females possess greater quantities of zinc (Zn), iron (Fe), chromium (Cr), and manganese (Mn) when compared to males. Muddasani et al. [18] and Dittmar et al. [12] examined the chemical profile of the keratinized nail matrix and the mineral composition of the nail plate. The study by Dittmar et al. [12] found females to have a higher sulfur content than males, but females exhibit a lower nitrogen–sulfur (N/S) ratio Despite higher sulfur levels in the female nail matrix, males possess more alpha-keratin disulfide bonds and a greater quantity of beta sheets in their nails at the amino acid composition level.It is hypothesized that the application of nail polish and cosmetic treatments (pedicures and manicures) on nails by females may block the alpha-keratin pores on their nails, which potentially impedes the formation of disulfide bonds [19].

Sex determination based on a nail’s chemical composition analysis rather than through a DNA approach is not very common. The most common instrumental techniques applied for human nails’ chemical composition analysis include Fourier transform infrared spectroscopy (FT-IR), Atomic Absorption Spectroscopy (AAS), Neutron Activation Analysis (NAA), Inductively Coupled Plasma Mass Spectrometry (ICP-MS), Laser-Induced Breakdown Spectroscopy (LIBS), Nuclear Magnetic Resonance (NMR), Raman Spectroscopy, Gas Chromatography (GC), Liquid Chromatography (LC), High-Performance Liquide Chromatography (HPLC), etc. [5,8,12,15,16,19]. All these techniques have been used for the classification of sex based on chemical composition differences in nail keratin proteins. To date, there has been only one study using fingernails for sex prediction without measuring a nail’s chemical composition [8]. The aim of our study is to assess the applicability of human nail samples for ATR FT-IR as an alternative to other methods for sex determination. Specifically, we are interested in whether the applied method has the potential to differentiate male and female donors based on their nail clippings.

Recently, optical spectroscopies, such as fluorescence, infrared (IR), and Raman Spectroscopy, have been used in forensic science but are still very new [10]. Over the past few decades, spectroscopic methods have become more popular since they can provide information on the chemical-to-molecular level and have comprehensively been used to investigate molecular structures, chemical bonds, and characterize the structural changes in biomedical tissues due to the numerous advantages of spectroscopic methods like little or no sample preparation, fast spectral measurements, their non-destructive nature, and their qualitative and quantitative analysis methods [5]. FT-IR spectroscopy is an established analytical tool that can be applied to identify both chemical and structural information from a sample. The infrared spectrum shows the unique vibrational characteristics of a sample based on the different absorption frequencies of functional groups [19]. Advanced FT-IR spectroscopic imaging can capture both spatial and spectral information simultaneously, which allows the chemical distribution across the sample area to be visualized. Furthermore, its non-destructive nature enables it to utilize the same portion of the sample for subsequent examinations [5,11]. Thus, ATR FT-IR is found to be a promising technique for non-destructive, rapid, quantitative, and qualitative methods, which are ideal properties for forensic analyses [5]. Nails have been studied to differentiate healthy and unhealthy individuals by identifying different trace elements in nails [18,20]. ATR FT-IR has been used for diagnosis and monitoring diabetes on fingernails [21,22,23]. Coopman R et al. [21]’s study hypothesized that a nail’s keratin proteins are prone to glycation, and an ATR FT-IR non-invasive diagnostic tool was used for assessing the glycation of nails. In Jurgeleviciene et al. [22]’s study, ATR FT-IR was successfully used to examine the glycation process in nail clippings. ATR FT-IR has been successfully used to differentiate chronic and acute diabetes mellitus in patient fingernail specimens [23]. Recently, ATR FT-IR has been successfully used in fingernails to evaluate chronic fatigue syndrome in patients. FT-IR spectral analyses have unveiled a reduction in the α-helix content, while an increase in the β-sheet content has been observed. This finding suggests an imbalance in the typical structural elements within the nail plate [24]. Another ATR FT-IR study on nail specimens has shown that the chemical penetration enhancer *N*-acetyl-l-Cysteine (a recognized penetration enhancer in healthy nails) perturbs the nail plate [17]. FT-IR spectroscopy has been used for protein secondary structural analyses based on the Amide I mode [5,17,24,25].

ATR FT-IR spectroscopy is becoming an interesting option for sensor research. Sensors that measure molecular fingerprints possess the ability to identify and quantify complex molecular information while maintaining inherent molecular specificity [26,27]. The FT-IR sensor is composed of an infrared light source that generates a sensing platform in which light–matter interactions occur. Mid-infrared radiation has been used mostly for fundamental research on molecular structures due to its high sensitivity and precise selectivity for molecular conformations [17,26,28]. In addition, ATR-FT-IR spectroscopy enables us to sense protein–protein interactions or ligand–receptor binding [28]. The IR sensing schemes are divided into two groups: direct sensors and indirect sensors [27]. Direct sensors detect optical property changes, whereas indirect sensors detect the chemical recognition processes of the sample [27]. A membrane-based ATR FT-IR sensor has been used to determine oil and surfactant indices in degreasing bath samples [29]. The ATR FT-IR sensor’s capability to detect conformational changes has made it a valuable tool for assessing the efficacy of anti-cancer drugs [25].

In this study, measured FT-IR nail spectra were used for a chemometric analysis to determine “Male” and “Female” sex from fingernail clippings. A machine learning (ML) algorithm was coupled to a nail clipping spectral analysis to predict and differentiate sex from 63 donor’s samples. ML algorithms make decisions by using previously obtained knowledge in a new situation without requiring programming at every step. As MLs are highly automated and self-modifying, they continue to improve over time as they receive new data. ML was developed to address multivariate, high-dimensional, and very complex real-world data problems [30]. To distinguish between female and male donors, first, a partial least-square discriminant analysis (PLS-DA, a discriminant analysis model) was implemented. Then, an artificial neural network (ANN) was applied for sex prediction and determination. Usually, an ANN acquires knowledge from a set of inputs, and based on these inputs, it fine-tunes the model parameters with respect to new knowledge. Thus, neural network processing is not based on any characteristics of a statistical distribution but works similarly to the human brain on the principles of pattern recognition and error minimization. Someone can think of this process as receiving information and learning from each experience so that certain patterns can be found in the data [31]. The previously applied PLS-DA model scores were used as input into the ANN model to reduce the computational burden and decrease the chance of overfitting. Thus, this proof-of-concept study investigates the performance of a non-linear ANN on PLS scores as compared to the popular chemometric method for classification, PLS-DA.

## 2. Materials and Methods

### 2.1. Sample Preparation

Human nail samples were collected from 63 volunteers of varied races and ages, comprising 37 males and 26 females. The samples consist of 1–10 donated nail samples from each volunteer, with a length of at least 1mm. The exclusion criteria for the donors were unhealthy nails showing distinct symptoms of skin or nail disease and the use of nail polish. All samples were cleaned first with 70% isopropyl alcohol. They were stored in zip lock bags and coded.

### 2.2. Spectra Acquisition

The spectra of samples were acquired with a Fourier transform infrared (FT-IR) spectrometer (Nicolet iS10, Thermo Scientific, Waltham, MA, USA) in the spectral range of 600–4000 cm^−1^. The resolution was set at 4 cm^−1^. The time required to scan each sample was set to 32 s; each sample was scanned 32 times; and the average spectrum was automatically taken. For each nail, spectra were acquired at various spots of the sample to consider the heterogeneity of the nail matrix. For each new sample, a background spectrum was acquired prior to collecting the spectral data and subsequently subtracted from all measured spectra. The OMNIC 9.8 software (Thermo Nicolet Corporation, Waltham, MA, USA) was used to control and manage the measurement. For every sample, the diamond crystal was cleaned with isopropanol and left to dry before further measurements. To ensure sufficient contact between the sample and the crystal, each nail sample was simply placed on the crystal and compressed using the ATR attachment. The spectral ranges of 600–1711 cm^−1^ and 2669–3800 cm^−1^ were used for further analyses as they showed the contribution from the biochemical composition of the samples.

### 2.3. Data Pre-Processing and Statistical Analysis

The OMNIC spectral (.SPC) files, with a total of 824 spectra, were imported into MathWorks MATLAB R2020b version 9.9.0.1570001 (Natick, MA, USA), supported by the Eigenvectors Research Inc. PLS Toolbox 9.0 (Manson, WA, USA), for further processing and the statistical analysis. PLS-DA modeling was performed using the PLS Toolbox 9.0 software. The ANN and ROC analyses were performed using R (4.2.2) software, the R package “neuralnet” [32], and the package “pROC” [33]. The spectra were subjected to preprocessing steps: the transformation of the transmission to absorbance (log(1/T)), a second-order derivative with a second polynomial, normalization by the total area, and mean centering. The preprocessing steps were first selected and applied on a training dataset only.

A hold-out (test) dataset of eleven samples (donors) was randomly selected from the group of 63 donors at the beginning of the analysis and used only at the end for external validation. The remaining 52 nail samples were used as the training dataset. In machine learning, there is no standard approach for the minimal sample size calculation in bio-spectroscopic studies [34]. A very popular systematic approach for the sample size calculation is the post hoc method of fitting a learning curve, which is a good indication of the amount of data needed to train a model [35]. Since the number of donors was already given, we focused on the training phase of the data analysis. The dataset was split in such a way that the training data was sufficiently large. The exact ratio depends on the data, but a larger portion of data in favor of the training data is typically optimal for small datasets. In our case, we selected a data split when the calibration model showed a lower variance during the training process, as has been shown elsewhere [5]. The samples were split into a calibration dataset (training) and a test dataset (external validation), approximately consistent with a 79:21 split, using donor-stratified random selection. Specifically, eleven donors (155 spectra) were randomly selected for the external validation (EV) and were moved to the test dataset, and the remaining 52 donors (669 spectra) were used for the training dataset before the statistical analysis.

### 2.4. Partial Least-Square Discriminant Analysis (PLS-DA)

An important aspect of any PLS-DA model is the determination of the number of latent variables (LVs) to ensure that the model includes only the variability important for prediction without introducing different types of noise [36]. Numerous different PLS-DA models were built with an increasing number of LVs, and an estimate of the classification error rate of each PLS-DA model was acquired via a single 10-fold venetian blind cross-validation (CV) on the training dataset. In the PLS-DA environment, the CV involved a series of ten steps, and in each step, 1/10th of the training dataset was moved out, and a sub-model was built using the leftover spectra. This model was used to predict the class assignment, i.e., “Female” or “Male”, on the moved-out spectra. The test set was then moved back into the calibration dataset, and another 1/10th of the spectra was used as the test set. This procedure continued until all the spectra were predicted. At the same time, every predicted spectrum went through this process only once. The number of LVs was defined during the CV of the PLS-DA so that the number of LVs that yielded the lowest classification error rate during the CV was selected to be used for the final PLS-DA model [5]. After successfully training and cross-validating the PLS-DA model, it was externally validated with the spectra from the eleven donors moved aside at the beginning of the data analysis. The PLS-DA method is easily interpretable and inherently a linear algorithm, capable of modelling only linear latent covariance [37]. However, biological data are often non-linear; thus, more complex non-linear machine learning methods may be more suitable for the analysis of biological data.

### 2.5. Artificial Neural Network (ANN)

Here, the method for tuning the neural network was the resilient backpropagation with weight backtracking method. The resilient propagation algorithm is a very popular method in backpropagation training due to its fast convergence speed [38]. The inputs were provided as LVs from the previously used PLS-DA method, and the output data classes were labeled as either “Female” or “Male”. Before a model is trained, several parameters must be successfully selected with an ANN.

To assess the prediction capacity of the final neural network, the model evaluation is expected to be accomplished using totally independent datasets. In this study, the datasets were divided into training and test sets, as described above. Further, cross-validation was applied to the training dataset for tuning the ANN parameters; thus, we did not have a separate validation dataset. The training process minimizes a training error, and the error is evaluated on the corresponding validation dataset. Numerous neural networks were built to tune the network architecture and parameters. Prediction on validation data provides an evaluation of a network model; thus, the hyperparameters of the model that gave the best results will be selected for the final model. A single 10-fold venetian blind cross-validation on the calibration dataset [39] is a good compromise in the sense of a bias–variance trade-off [40]. Once the ANN hyperparameters, together with the network architecture (the number of layers and neurons), were validated through their performance on the CV datasets, the final network was used to predict the labels of the unknown samples in the hold-out dataset.

## 3. Results

The aim of this work was to explore the applicability of ATR FT-IR spectroscopy as a suitable method for male and female donor differentiation based on human nail clippings. The most appropriate project design was selected based on our preliminary results. Human nail samples make excellent specimens. They make a reservoir substance out of tightly bound keratin fibers. The matrix of nail plates is porous, making it a superb matrix for trapping different biomarkers and analytes. Nail specimen collection is a noninvasive and simple process, and the nails only need to be stored in a dry place at room temperature. The ATR FT-IR spectra collected from the male and female nail samples showed similar spectral patterns. To determine the donor sex from the human nail spectra, multivariate discriminant models were built using the PLS-DA algorithm and the ANN algorithm. The ANN algorithm increased the discrimination performance of the model and identified the spectral origins for sex discrimination.

### 3.1. Average Mean Raw Spectra of Nails

**Figure 1:**
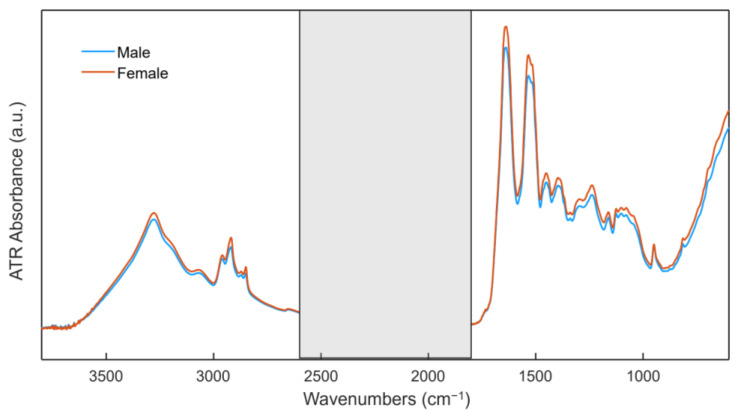
Averaged raw spectra of nail clippings for the groups of males (blue line) and females (red line). The spectral ranges from 600 to 1711 and 2669 to 3800 cm^−1^ showed contributions to the final spectra and were used for further analysis. Spectral regions from 1711 cm^−1^ to 2669 cm^−1^ (grey rectangle) were excluded from the analysis to avoid interference from the diamond ATR crystal.

The average ATR FT-IR spectra of the two different groups based on human nails are presented in Figure 1. The average “Male” and “Female” ATR FT-IR spectra are very similar, characterized by the same bands of similar intensities. Since the basic components of human nails are similar both in males and females, we look for every minute difference in composition based on sex. The visual representation clearly (Figure 1) shows that it is quite impossible to differentiate two groups for our purpose; therefore, it necessitated an advanced multivariate analysis in our case. this proof-of-concept study did not include a chemical analysis of the nails, we applied advanced ML techniques to acquire nuance pattern differences to build the prediction models to differentiate gender in the unknown samples. The spectral signature of nails reveals vibrational frequencies associated with various biomolecules and provides valuable insights about the essential components present in human nails. The nail IR spectra can be well described by major regions assigned to proteins, but they also show contributions from nucleic acids (1000–1250 cm^–1^), lipids (2800–3000 cm^–1^), and carbohydrates (1000–800 cm^–1^). The peak assignment of molecular vibrations for human nails is available in the literature from our previous study [5].

The primary component of nails is keratin, a fibrous protein. Keratin can be classified into alpha-keratin and beta-keratin. In the literature, there are other classifications being used, where keratin can be classified as soft or hard keratin. The amount of sulfur and lipids in soft keratin is lower. Nail keratins are hard keratins, where the amount of sulfur is higher [41]. Many studies have been conducted on the chemical analysis of nails to discriminate between “Male” and “Female”. Our study focus was to apply a multivariate analysis using ATR FT-IR spectral data to acquire subtle spectral pattern differences between “Female”- and “Male”-originated samples.

**Figure 2:**
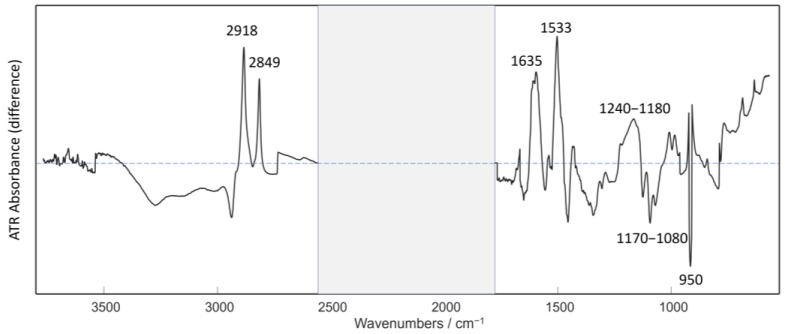
Difference spectrum of the calculated normalized average spectra of the “Female” class and the “Male” class. Absorption peak intensities show the differences between the male and the female groups; peak intensities above the line represent “Female” and below represent “Male”. The horizontal blue dashed line represents the zero line. The grey shaded region represents the spectral range excluded from the analysis.

Although the spectral profiles appear similar, a difference spectrum of the calculated mean spectra of the “Male” and “Female” classes shows some distinct features. The differences could be associated with the ATR FTIR spectral band assignments based on the human nails available from our previous study [5]. In our previous study, we showed the assignment of the main characteristic bands of the FTIR spectra of human nails. They confirmed the presence of keratin proteins as the main component, as well as lipids and nucleic acids. As highlighted in Figure 2, the biggest differences in the intensity of the characteristic bands between the “Female” and “Male” classes are for lipids and proteins at 2920 cm^−1^ and at 2849 cm^−1^, for Amide I at 1635 cm^−1^, and for Amide II at 1533 cm^−1^, showing a higher intensity of bands in the female class. The Amide I band is typically a very intense band in proteins, and differences found in this band are directly related to the backbone conformation [42]. The largest difference was observed in the Amide II band at 1533 cm^−1^, which is a rather complex band, so the identification of changes in conformation is very complicated. Slight intensity changes were also revealed in the region 1240–1180 cm^−1^, which corresponds with the Amide III band, C–N stretching vibrations, and the PO_2_^−^ asymmetric stretching of nucleic acids. Additionally, a small intensity variation has been observed in the spectral region 1170–1080 cm^−1^, corresponding to the PO_2_^−^ symmetric stretching of nucleic acids. The peak maxima of the difference spectrum correspond to the absorbance bands of the female nails, whereas the peak minima are associated with the male nail spectra.

### 3.2. Partial Least-Square Classification (PLS-DA)

**Figure 3:**
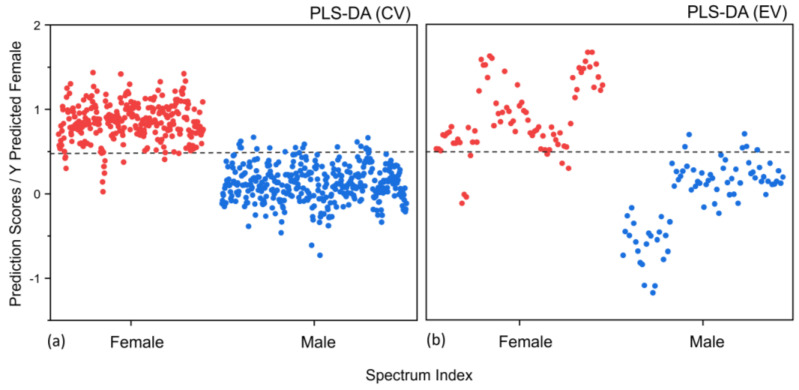
(**a**) Calibration (CV) and (**b**) external validation (EV) prediction results for “Male” and “Female” based on the human nail clippings. Each dot represents the individual ATR FT-IR spectrum data for male and female nails. The red dot represents “Female”, and the blue dot represents “Male”. The broken dotted line denotes the classification threshold.

The pre-processed spectral data were initially subjected to a PLS-DA analysis for dimensionality reduction, and 12 latent variables (LVs) were included for further analysis as they were considered to contain enough information to explain the variance in the spectral data. In a PLS-DA, LVs are calculated as a linear combination of the associated original variables rotated to maximize their relationship with a response variable. The PLS-DA model was designed using the FT-IR spectra of males and females with 12 LVs and was validated through 10-fold venetian blind cross-validation (CV), as described above. Twelve LVs were determined during the CV when ten subsets of spectra were moved aside, and their class assignment was predicted. The CV results were acquired as the averages of sensitivity (true positive rate female) and specificity (false positive rate male) and total discriminant accuracy. We achieved a sensitivity of 96% and a specificity of 95% with 95% accuracy to differentiate between the male and female donors from their nail clippings. After the PLS-DA calibration model was trained with the twelve LVs for the “Male” and “Female” differentiation of nail specimens, we could predict spectra from the test set that were never seen by the model during the calibration. This allowed us to determine an unbiased evaluation of the final PLS-DA model. Figure 3a shows the discriminant scores predicted for each individual nail spectrum through the CV process, and Figure 3b shows the discriminant scores predicted for each individual nail spectrum through external validation (EV). Figure 3 shows the prediction scores for each spectrum and the classification threshold (grey dashed line). Any spectrum found above the threshold line is predicted as “Female”, and any spectrum below the classification threshold line is assigned as the “Male” spectrum.

The score plot in Figure 3b indicates that the PLS-DA model effectively worked to distinguish the test data of each sex class into a specific range of scores. Any spectrum located above the threshold is predicted to be “Female”, and any spectrum below the threshold is predicted to be “Male”. However, some of the individual spectrum data were misclassified (six male and seven female spectra) into incorrect classes. The external validation (EV) on the test dataset resulted in a sensitivity of 91% and a specificity of 92% with an accuracy of 92% to discriminate between the “Male” and “Female” classes. On the donor level, the PLS-DA model showed an accuracy of 100% for differentiating between the two groups when a standard 50% threshold was used. Each donor was classified as the sex that received the most assignments (>50%).

**Figure 4:**
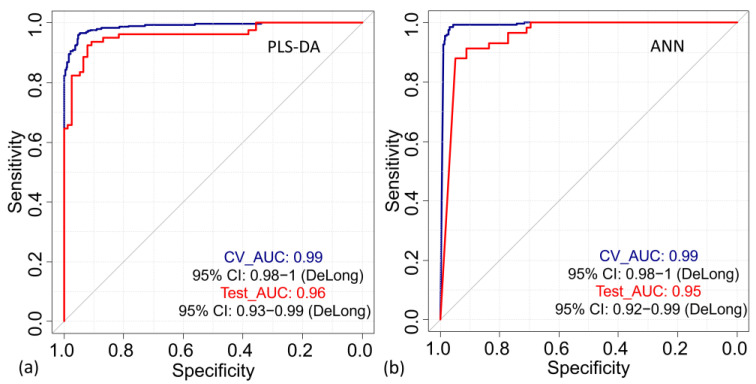
Comparing specificity versus sensitivity across different decision thresholds to assess a dichotomous outcome of the PLS-DA model (**a**) and ANN model (**b**) presented as a receiver operating characteristic (ROC) curve. In the PLS-DA model, the area under the ROC (AUROC) achieved was 0.99 (95% confidence interval (CI), 0.98–1) and 0.96 (95% confidence interval (CI), 0.93–0.99) during cross-validation (CV) and external validation (EV), respectively. In the ANN model, the area under the ROC (AUROC) achieved was 0.99 (95% confidence interval (CI), 0.98–1) and 0.95 (95% confidence interval (CI), 0.92–0.99) during CV and EV, respectively. Such results show that the “Female” and “Male” groups are well separated for both the PLS-DA and ANN classification models.

Characteristic (AUROC) values indicate the probability that the discriminant model can correctly classify the nail spectral data of each sex class. Figure 4a shows the performance of the classification PLSDA model presented as an ROC curve plot, which shows how sensitivity and specificity change depending on different thresholds. The obtained AUROC value is 0.99 (95% confidence interval (CI), 0.98–1) and 0.96 (96% confidence interval (CI), 0.93–0.99) for the CV and the EV, respectively, indicating that the female and male groups are well separated.

### 3.3. Artificial Neural Network (ANN)

The use of a supervised ANN is a technique that relies on prior knowledge about the class assignment of all the spectra in the training dataset. The ANN needs to first be calibrated on the training dataset. The scores from the PLS analysis were introduced to the model through the input layer to allow one or more hidden layers to perform the data processing through weighted connections between neurons and the output layer to give the classification results. A total of 669 labeled spectra were introduced to the network. To calibrate the model, the network weights need to be adjusted to minimize the training error. As mentioned before, this process of weight optimization was based on the resilient backpropagation algorithm with weight backtracking with a threshold set to 0.01 described for the partial derivatives of the error function [32]. The error function was selected as the cross-entropy error (CEE) that is recommended for classification problems. To find the best network for our training dataset, different parameters must be optimized. Hyperparameter tuning is a purely empirical process, so determining the number of hidden layers, the number of neurons in each hidden layer, and tuning other hyperparameters of the network algorithm requires testing many possibilities. All the created neural networks predicted the class membership for the spectra of unknown labels in the validation datasets during the CV (venetian blind CV) to determine the optimal ANN to differentiate the female and male donors [43]. Choosing the correct model with appropriate parameters is crucial for accurate predictions of the sexes of the nail donors. The neural network structure that yielded the best CV results with the lowest prediction errors for the validation datasets was a three-layer neural network with one input layer, one hidden layer with five neurons, and an output layer. The initial weights of the network were randomly initialized. When the final ANN with appropriate parameters was identified, the network weights were fixed.

**Figure 5:**
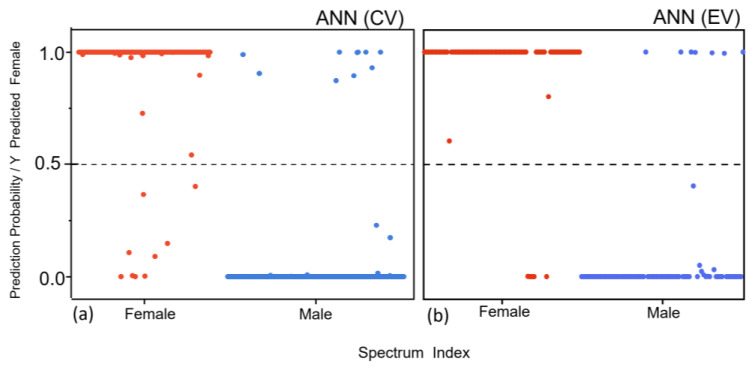
Results of the final predictions of the ANN model showing the estimated classification probability for each spectrum. All spectra were scored with the probability with which the spectrum is assigned as “Female” from the venetian blind CV (**a**) and from the external validation (**b**) of the ATR FT-IR spectra of nail samples. The calculated probability for each spectrum was classified as either “Female” (when above 50% threshold) or “Male” (when below 50% threshold). The probability for the correct assignment of an individual ATR FT-IR nail spectrum is marked with colors as “Female” (red dots) and “Male” (blue dots). The broken dotted line denotes the 50% classification threshold.

We trained our ANNs by minimizing the cross-entropy error (CEE) [44]. For all the validated spectra during the CV, the classification results were calculated as the probabilities of the spectra being assigned as female. For ten validated subsets from all 52 donors (669 spectra), the “Female”/“Male” class predictions and associated probabilities were reported for each spectrum throughout the CV. Figure 5a shows the ANN results for the female vs. male FT-IR spectra binary classification, as determined through the venetian blind CV. In all 10 validation sets, 19 spectra were misclassified, predicting the spectrum of a female as a male, and vice versa. The threshold value was set by default to 50%. The achieved sensitivity, specificity, and accuracy were 97% for all the parameters. The final network achieved a performance, in terms of the AUC performance, on the validation dataset of 0.99 (95% confidence interval (CI), 0.98–1). On the donor level, the ANN model showed 100% accuracy for differentiating between the two groups when the standard 50% threshold was used, which means no donor was misclassified. Once an ANN was trained and validated, an EV on the hold-out dataset was performed. Here, 155 spectra, originating from eleven donors, were already separated before the statistical analysis. To end up with the points from the training and test datasets in the same space without using any knowledge about the test dataset during the training, first the PLS-DA analysis was performed on the training set, and the obtained LVs were saved and then used to transform the points in our test set.

The EV of the ANN model on the test dataset resulted in a sensitivity of 93%, a specificity of 91%, and an accuracy of 92% to discriminate between the two classes. The donor-based classification results showed a 100% accuracy rate to discriminate the donors class. All the donors from the test dataset were correctly classified. The obtained AUROC value is 0.99 (95% confidence interval (CI), 0.98–1) during the CV and an AUROC of 0.95 (95% confidence interval (CI), 0.95–0.99) in the EV in differentiating between “Females” and “Males” using human nails as substrate.

## 4. Discussion

To distinguish between the male and female donors from the nail clippings, the PLS-DA model and the ANN non-linear model were implemented. We tested several techniques on our spectral data to select the most suitable algorithm for our particular problem. Choosing the correct model with the appropriate parameters is crucial for accurately predicting the sex of a nail donor.

The PLS algorithm is a linear regression method that may be used for classification. When the PLS algorithm is used with a dummy response variable, the PLS model is called the partial least-square discriminant analysis (PLS-DA) model [45]. The extra step in the PLS-DA model is a thresholding of predicted y-values to assign class labels to a spectrum (females vs. males). The PLS algorithm is a linear method that is responsible for reducing the dimension of the data (i.e., the full FTIR spectrum) into a few alternative axes to efficiently separate the data of each group [46]. The new PLS components’ latent variables (LVs) are selected in a supervised manner to minimize the influence of irrelevant variables and maximize covariance between the responses (female and male class assignment) and a new linear combination of the original features. Then, the final dimensionality (the number of the LVs) is resolved by deciding how many of these new predictors will be included in a model.

Here, our primary objective for pattern recognition was to construct an effective classification model. Building a classifier based on ATR-FTIR spectral data can be carried out for various purposes, such as for a screening tool to determine alcohol presence, gender determination, phenotype profiling, evaluating the authenticity of any substances, etc. [47]. There are many chemometric classification models available these days, including principal component analysis (PCA), partial least-square discriminant analysis (PLS-DA), support vector machines (SVMs), random forests (RFs), logistic regression (LR), and artificial neural networks (ANNs). Numerous citations in the Chemical Abstract Database highlight that these classification models exhibit the potential to effectively handle extensive sets of high-dimensional multivariate data with varying qualities, leading to reliable predictions [48,49].

The PLS-DA and PCA both are quite popular methodologies for dimensionality reduction. Hence, PLS models are often better at capturing information relevant to the given problem than a corresponding PCA model [50]. PLS has become a very popular tool for multivariate data analyses because of the good performance of the calibration models produced and how easy it is to implement the method, given the wide availability of PLS software [51]. In the case of PLS models, the development of latent variables (LV) takes place simultaneously with model calibration. In PLS models, the issue of confounding and unwanted factors interfere with the desired signal, is typically less problematic because a PLS model employs an iterative process that considers both the response and the measurement variables to determine the PLS components. There is a complex relationship between nail-metabolic fingerprinting and environmental/behavioral factors, as we have already shown in our previous work [5]. In this study, a similar situation is reflected in the fact that the final model complexity, i.e., the number of LVs, was estimated to be twelve for the differentiation of the two groups of nail specimen donors.

Despite having a successful classification rate from the PLS-DA model for sex determination, we additionally built the ANN model to further validate and enhance the accuracy of the sex determination process. ANNs have confirmed their ability to describe non-linear relationships well, which has proven to be very useful, especially in recognizing patterns and performing forecasting on spectroscopic data [43,46,52,53]. Artificial neural networks (ANNs), inspired by the biological interconnections in the brain, can be described as several layers of simple, weighted, interconnected mathematical operators called neurons. Each neuron acts as a weighted sum of the outputs of the previous layer applied to an activation function (typically a linear or logistic function). ANNs are composed of several layers: the input layer, one or more hidden layers that perform processing through a system of weighted connections, and the output layer, which finally gives the classification result [48].

During the training process, the neural network learns the relationship between the independent and dependent variables. Specifically, it may learn to associate varying intensity values of specific wavelengths of the FT-IR spectral data with sample class assignments, “Female” and “Male” labels. However, as the number of original input variables increases, the number of weights in the network increases. Consequently, the amount of data needed to determine the weights of the network also increases, and overfitting can become an issue. A dimensionality reduction can reduce the size of the network and, hence, the amount of data needed to train a model. The scores from the previously used PLS-DA method can be used as an input for an ANN. This approach will ensure that representative spectral LVs will be selected, and the nonlinearity between the spectral measurement and the sample class membership will be effectively handled by the ANN [49].

Most neural networks are trained with supervised training algorithms. For an ANN, this means that the network processes the known inputs and compares its actual outputs against the expected outputs. The errors are then backpropagated via the network, and the weights are adjusted considering the errors returned. This process is repeated until the errors are minimized. The weights between the individual layers during this training are gradually updated, so the same dataset is processed many times (number of iterations) for the sake of learning. This supervised learning algorithm, which is very popular, is often referred to as a backpropagation algorithm (backward propagation) [45].

ANNs are highly complex systems, which contribute to their efficiency, robustness, fault tolerance, and resistance to noise. ANNs can learn from training data and generalize their knowledge to handle new unknown data in previously unseen situations. Moreover, an ANN provides remarkable information-processing characteristics such as non-linearity, which allows it to better fit the data, and noise insensitivity, which provides accurate predictions in the presence of uncertain data and measurement errors. When an ANN is given sufficient input data, it can learn through successive training and predict a specific outcome. In this study, the original FT-IR spectral data were assigned as input values for the PLS-DA algorithm, labelled as “Male” or “Female”. The PLS-DA scores, which represent the original data in a lower-dimensional subspace, were used as input variables for the ANN classification model. At the 50% threshold value, we obtained sensitivity, specificity, and accuracy of 93%, 91%, and 92%, respectively, at spectral-level classification. On the donor-level classification, we achieved 100% accuracy for differentiating between the two groups based on an independent dataset during the external validation.

The PLS model is easily interpretable and is inherently a linear algorithm. It is only capable of modelling linear latent covariance. Since our samples, nail data, are heterogenous, we hypothesized that a more complex non-linear machine learning method would be more suitable for the analysis of biological non-linear data. This hypothesis was supported by our results. The PLS-DA was able to select the correct hyperplane on the samples, achieving high accuracy, but the separation between the clusters was lower (the values were close to the classification threshold). Even though the accuracy is almost the same for both methodologies, the separation between the predictions of individual spectra, whether they are females or males, is significantly greater in the case of the ANN, both in the CV outcome and in the EV outcome.

Here, we demonstrated a novel method for determining a donor’s sex based on the ATR FT-IR spectroscopy of human fingernails and a chemometric analysis using ANNs applied to PLS-DA scores. The results strongly indicate the potential of this developed method for donor sex determination in the forensic analysis of human nails. The established model was found to give a better score–space separation between the two sex groups of human nail spectra when an ANN was used on the PLS-DA scores. Additionally, compared to the previously published results on sex screening from human nails based on FT-IR spectra [8], our developed model clearly improved the separation between the groups. It should also be mentioned that the results of a statistical analysis in the previous study did not generalize to an independent dataset [5].

## 5. Conclusions

There is a high demand in forensic science for analytical methods that are rapid, easy-to-use, inexpensive, and non-destructive, with selective capabilities that would make them ideal for the presumptive or confirmatory testing of forensic evidence. Advances in instrumentation, innovative algorithm development, the proficient handling of large data, and computing resources are developing fast. The incorporation of multivariate methods into forensic analyses is increasing tremendously as it helps in deciphering all the aspects of an investigation, such as the identification, differentiation, and classification of trace evidence. Despite the momentary limitations in forensic practical applications, it clearly endorses the recent developments of different sensors for future applications in the forensic field. Vibrational spectra reflect information about the overall molecular composition of a sample. Mid-infrared radiation (MIR) spectroscopy is the FT-IR spectroscopic method of choice when analyzing biological materials since it covers the fundamental vibrational modes of important biomolecules. The combination of vibrational spectroscopy and advanced statistics provides high potential virtually for any industry and area of applications, such as rapid diagnostics in clinical settings, environmental tests, measurements of food quality, forensic applications, etc. Our technique for sex determination from human nails based on ATR FT-IR spectra combined with an ANN approach can be applied to narrow down a list of suspects with reliable information and potentially provide a novel strategy to resolve crimes. The readiness of the portable ATR FT-IR instrument allows for the in situ examination of samples, thus further increasing the potential of this novel approach.

Here, we demonstrate the development of a novel method of ATR FT-IR spectroscopy coupled with a machine learning analysis. The IR spectrum shows the specific vibrational characteristics of a sample that originate from the different absorbance frequencies of the functional groups [5]. The spectral features found through ATR FT-IR showed the presence of prominent Amide A and B bands in the fingerprint region. Our previous study confirmed the presence of the Amide I, Amide II, and Amide III bands through feature selection using an sPLS model, which indicates the presence of keratin proteins along with other hydrocarbon groups from lipids to proteins [5]. ML algorithms can reveal spectral complexity information, including spectral compositions and the minute differences caused by donors’ sexes, “Male” and “Female”. Both the PLS and ANN models successfully differentiated between “Male” and “Female” from the nail clipping samples. Both the PLS-DA and ANN models showed, during the external validation, an excellent AUROC value of 0.96 and 0.95, respectively, to classify the “Male” and “Female” groups. The established ANN model was found to give a better score–space separation between the two sex groups though. Thus, the aim of our work was to examine the practicality of human nail samples combined with ATR FT-IR as a replacement method for those methods currently used for gender determination. Specifically, we were interested in whether the applied method has the potential to differentiate between “Male” and “Female” donors based on their nail clippings.

This study has shown that FT-IR spectroscopy coupled with multivariate classification techniques can be a very useful analytical method for sex classification. Furthermore, the promising results from this study make it possible to explore additional analytical applications for other personal characteristic classifications in which the classes to be differentiated show significant internal variations, such as age or race.

## Data Availability

The data that support the findings of this study are available on request from the corresponding author.
